# Mindfulness-Based Program for Anxiety and Depression Treatment in Healthcare Professionals: A Pilot Randomized Controlled Trial

**DOI:** 10.3390/jcm10245941

**Published:** 2021-12-17

**Authors:** Mirian Santamaría-Peláez, Jerónimo Javier González-Bernal, Juan Carlos Verdes-Montenegro-Atalaya, Luis Ángel Pérula-de Torres, Ana Roldán-Villalobos, Esperanza Romero-Rodríguez, Nur Hachem Salas, Rosa Magallón Botaya, Teresa de Jesús González-Navarro, Raquel Arias-Vega, Francisco Javier Valverde, María Jiménez-Barrios, Luis Alberto Mínguez, Benito León-del-Barco, Raúl Soto-Cámara, Josefa González-Santos

**Affiliations:** 1 Department of Health Sciences, University of Burgos, 09001 Burgos, Spain; mspelaez@ubu.es (M.S.-P.); mjb0007@alu.ubu.es (M.J.-B.); rscamara@ubu.es (R.S.-C.); 2 Family and Community Medicine Teaching Department of Burgos, 09006 Burgos, Spain; juancarlosverdesm@yahoo.es; 3 Multi-Professional Teaching Unit for Family and Community Care of Córdoba, Healthcare District of Córdoba and Guadalquivir, Institute Maimónides of Research Córdoba (IMIBIC), Reina Sofía University Hospital, University of Córdoba, 14001 Cordoba, Spain; langel.perula.sspa@juntadeandalucia.es; 4 Carlos Castilla del Pino Health Center, Healthcare District of Córdoba and Guadalquivir, Institute Maimónides of Research Córdoba (IMIBIC), Reina Sofía University Hospital, University of Córdoba, 14001 Cordoba, Spain; aroldanvi@gmail.com; 5 Healthcare District of Córdoba and Guadalquivir, Institute Maimónides of Research Córdoba (IMIBIC), Reina Sofía University Hospital, University of Córdoba, 14001 Cordoba, Spain; emromerorodriguez@gmail.com; 6 Mediterráneo-Torrecardenas Health Center, 04009 Almería, Spain; nurhachem@gmail.com; 7 IIS-Aragon—Group B21-R17, Family and Community Medicine Teaching Department of Zaragoza Sector 1, Institute of Health Carlos III—REDIAPP 06/18, University of Zaragoza, 50018 Zaragoza, Spain; med000764@gmail.com; 8 Emergency Service, University Hospital of Torrecardenas, 04009 Almería, Spain; teresagonzalezn@gmail.com; 9 Castello Health Center (Madrid), Institute Maimónides of Research Córdoba (IMIBIC), Reina Sofía University Hospital, University of Córdoba, 14001 Cordoba, Spain; mrav98@gmail.com; 10 Family and Community Medicine Teaching Department of Jaen, 23007 Jaen, Spain; franciscoj.valverde.sspa@juntadeandalucia.es; 11 Department of Educational Sciences, University of Burgos, 09001 Burgos, Spain; laminguez@ubu.es; 12 Department of Psychology, Faculty of Teacher Training College, University of Extremadura, 10071 Caceres, Spain; bleon@unex.es

**Keywords:** anxiety, depression, mindfulness, MBSR, primary care, mentors, resident intern specialists

## Abstract

In primary health care, the work environment can cause high levels of anxiety and depression, triggering relevant expert and individual change. Mindfulness-Based Stress Reduction (MBSR) programs reduce signs of anxiety and depression. The purpose of this sub-analysis of the total project, was to equate the effectiveness of the standard MBSR curriculum with the abbreviated version in minimizing anxiety and depression. This randomized controlled clinical trial enrolled 112 mentors and resident specialists from Family and Community Medicine and Nurses (FCMN), distributed across six teaching units (TU) of the Spanish National Health System (SNHS). Experimental group participants received a MBRS training (abbreviated/standard). Depression and anxiety levels were measured with the Goldberg Anxiety and Depression Scale (GADS) at three different time periods during the analysis: before (pre-test) and after (post-test) participation, as well as 3 months after the completion of intervention. Taking into account the pre-test scores as the covariate, an adjusted analysis of covariance (ANCOVA) showed significant depletion in anxiety and depression in general (F (2.91) = 4.488; *p* = 0.014; η^2^ = 0.090) and depression in particular (F (2, 91) = 6.653; *p* = 0.002; η^2^ = 0.128 at the post-test visit, maintaining their effects for 3 months (F (2.79) = 3.031; *p* = 0.050; η^2^ = 0.071—F (2.79) = 2.874; *p* = 0.049; η^2^ = 0.068, respectively), which is associated with the use of a standard training program. The abbreviated training program did not have a significant effect on the level of anxiety and depression. The standard MBSR training program had a positive effect on anxiety and depression and promotes long-lasting effects in tutors and resident practitioners. New research is needed to demonstrate the effectiveness of abbreviated versions of training programs.

## 1. Introduction

The level of health perceived by workers is closely related to the psychosocial components of the work environment. These components directly affect a person’s professional life, harming their physical and mental health, as well as the quality of life, contributing to the emergence and manifestation of various pathologies [1].

The psychosocial environment of health professionals and especially primary health care personnel is characterized by a high degree of self-perceived stress and tremendous emotional and psychological demands. For this reason, these experts have a greater risk of developing anxiety and depressive disorders than the rest of the population [2,3]. In addition, the coronavirus pandemic has had a major impact on both these health experts’ quality of life and psychological health [4].

Some of the stressors are inherent in the health profession, such as long working hours, unpredictable work, dealing with pain, suffering, and death, or supporting families. However, there are other external components that can increase the degree of stress experienced by professionals, such as high load of work, staff shortages, and patients who are continuously asking for solutions for their problems and inconveniences, the greatest need for understanding, insufficient time to continue learning and recycling their professional specialty, or a feeling of poor support from their managers [5,6,7]. In addition, as a side effect of the pandemic coronavirus disease, fear of infection, the likelihood of transmitting the disease to loved ones, confinement, and even voluntary isolation increase the likelihood of stressful situations [8,9].

Regarding stress, the main effects on health professionals are burnout, anxiety disorders, and lateral violence [10]. Previous studies have shown that health care activities involve inherent stress that can damage high levels of cognitive functions, particularly attention and memory, and lead to anxiety and depression, with relevant individual and professional effects, including a decrease in patient satisfaction, decrease in job satisfaction, increase in medical errors, interpersonal interaction disruption, substance abuse, and other mental health problems [11,12,13,14,15,16].

Depression is a mood disorder that is accompanied by a loss of interest and a continuous sadness feeling. Several studies have reported a positive significant correlation between the occurrence of signs of anxiety and mood and the level of job stress. In addition, there is also an important association between depressive disorders and other relevant health conditions, particularly chronic ones, such as a somatization disorder, chronic fatigue, and psychotropic drug consumption [17,18,19,20].

Some researchers have shown the need of interventions to improve primary care specialists’ psychological health, especially during the early stages of pandemic coronavirus disease [21,22]. Once mindfulness training programs were compared with active control conditions, they proved to be a useful technique to reduce perceived stress and signs of anxiety and depression in health experts, improving different indices of psychological health and comfort, including quality of life, chronic pain, or emotional distress [23,24,25,26,27,28]. The results of a meta-analysis that included 209 clinical trials by Khoury et al. showed that mindfulness-based training was more effective in minimizing the severity of patients’ signs of anxiety and depression than the waiting list, psycho-education, therapeutic support, relaxation techniques, and imagery or suppression procedures. In addition, after a follow-up period of between 3 weeks and 3 years, and a median of 28 weeks after meditation, it was observed that the effects obtained after the mindfulness-based training program, remained over time [29]. Additionally, this class of interventions works through changes at specific points in psychopathology, such as cognitive function, emotional deregulation, and interpersonal effectiveness [25,30,31].

Kabat-Zinn defined mindfulness originally in 1979 as the ability of paying attention to the end in the present moment without evaluating the development of one’s own experiences from moment to moment [32]. This type of meditation practice is based on attention’s self-regulation and consciousness in order to enhance mental processes’ control and increase personal well-being [33,34].

Self-compassion is understood as the function of responding to oneself in moments of failure or discomfort with instruction, decency, and understanding. This aspect is important for health professionals because they need to know how to respect and confess themselves in order to convey these emotions to the people to whom they provide their services. Self-compassion is an element of resilience associated with less psychopathology, stress, and greater comfort. It is related to mindfulness and is constantly included in training programs in order to improve the interaction between doctor and patient [35,36].

The MBSR training program, developed by Kabat-Zinn [37], is made up of eight group sessions per week lasting 2.5 h all together, and 45 min of practice at home, 6 days per week. The results of various meta-analyses on this program have shown useful results for mental and physical health in various clinical populations [38,39]. However, this program requires a significant amount of time to complete the training, which is an obstacle for many people and limits its use in many situations [40]. Various investigations have tried to lessen the duration of these programs to 4 weeks for maintaining their effectiveness. In a systematic review, an abbreviated MBSR program was shown to be as effective as the standard training for maintaining the psychological management of health professionals. However, the samples of the different studies with which the abbreviated four-week MBSR program was implemented were reduced (16–20 participants), and none of them were mentors and resident specialists in Family and Community Medicine and Nurses (FCMN) [23].

The Spanish National Health System (SNHS) adopted the residency system for the postgraduate education of health specialists, such as Medicine and Nursing. This system is made up of Teaching Units (TU), which are the set of human resources and support materials, teaching, and research, necessary for regulated training in Health Sciences disciplines, regardless of their ownership. After passing an annual national examination, licensed doctors and nurses have the possibility to complete a training period, ranging from 2 to 5 years, to become specialists in a special area of Medicine or Nursing; during this period, they are called resident internal specialists. Along the training process, they are expected to participate and progressively assume responsibilities in the diverse areas of competence of their own specific specialty program. The mentor is a key figure in this process. He is a professional with high experience and knowledge about patient care and he altruistically and voluntarily supervises resident internal specialist duties and activities [41,42,43]. Consequently, mentors and resident in-house specialists share responsibilities and expectations for education and learning similar to clinical practice. There are few studies conducted providing enough evidence on the effectiveness of the MBSR abbreviated interventions in health specialists, especially in mentors and resident specialists in FCMN. Therefore, the objective of this sub-analysis was to analyze the effectiveness of two MBRS programs, an abbreviated and a standard one, on anxiety and depression in mentors and resident specialists in FCMN.

## 2. Materials and Methods

### 2.1. Study Design

The Mindfulness Teaching Units (MINDTU) study was a controlled multicenter empirical, cluster-randomized, open-label, pragmatic study of equal effectiveness with 3 parallel groups [44]. Its goal was to establish the impact of mindfulness and self-compassion program (MBRS) on mentors and resident FCMN professionals’ burnout and work stress.

The MINDTU clinical trial protocol specifying data collection procedures was previously disclosed [44] and also registered with the ClinicalTrials.gov web portal maintained by the US National Library of Medicine under ID number NCT03629457.

In this paper, a sub-analysis of participants’ levels of anxiety and depression is shown as part of secondary results from a clinical trial.

### 2.2. Recruitment and Participants

This research was developed in Primary Care, and participants were selected in the next 6 Teaching Units (TU) of the SNHS: Almería, Burgos, Córdoba, Jaén, Ponferrada, and Zaragoza Sector I. The TUs were selected according to the population density of each territory, distributed across the Spanish geography. All mentors (*n* = 297) and internal specialist residents (*n* = 595) in FCMN at these units constituted the population under analysis. Eligibility criteria included being active and signing the informed consent form after receiving analysis data. Individuals who had followed a mindfulness module or seminar of minimum 4 weeks, who frequently practiced mindfulness techniques, were on long-term sick work leave due to pathology, or had a long-term illness, or with mental disorders that discourage the development of interventions were excluded.

The analysis was disseminated through the communication options that exist in all 6 TUs. The likely participants were recruited through a face-to-face meeting of 1 h, where the objective, methodology, and voluntary nature of the analysis were described. They were invited to participate in the analysis, filling out, and signing the commitment term and the free and informed consent form.

### 2.3. Sample Size

The feasible mean modification in Mindfulness Five Facet Questionnaire (FFMQ) score, as the principal variable in this analysis, were used for the sample size estimation a priori. It was assumed a 0.05 alpha risk, a 0.20 beta risk, and a standard deviation (SD) of ± 20 points in bilateral contrast, with an expected drop-out rate of 25% throughout follow-up. There was a requirement of 140 participants (38 in each group) in order to detect the minimum difference of ≥15 points in the FFQM score of the experimental groups (EG) when they were compared to the control group (CG). The results of previous similar investigations were used to calculate this estimation [45,46]. In addition, the effects of the analysis type of its design were further examined once the sample measurement was calculated. Therefore, to obtain the same power between intra and intergroup variance, a multiplication factor of 1.7 was applied, assuming a cluster size of 15 and an intragroup correlation coefficient < 0.05, similar to the most common in randomized clinical trials in Primary Care [47,48]. Assuming this value, 132 subjects, 44 in each group, and 22 for each TU, were considered sufficient to detect clinically significant differences in the primary variable of the study.

### 2.4. Procedure and Randomisation

**Figure 1:**
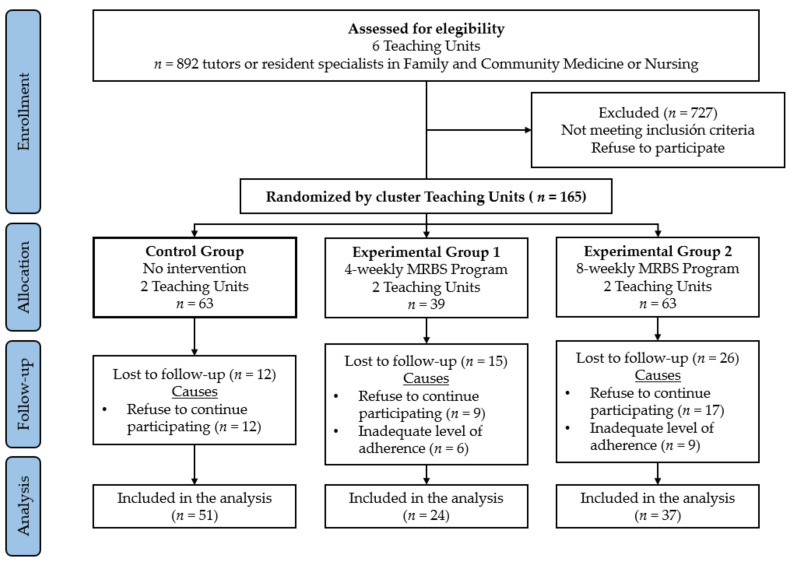
Flow participants’ chart through the research.

The assessment and measurements of the participants were taken at three time points. In the baseline or initial evaluation visit (pre-test) carried out one week before the interventions began, the study variables of all participants from the different groups were collected. Consequently, in the final evaluation (post-test), all participants were assessed again which took place 8 weeks after the baseline evaluation for those of the CG and EG2 participants and 4 weeks for the EG1. Furthermore, EG1 and EG2 participants were reassessed in the follow-up visit, 3 months after the intervention had finished, in order to verify the conservation or not of their outcomes over time. At the beginning of the study, there were 892 professionals available to participate; however, since many of them (*n* = 727) did not meet the inclusion criteria required to participate in the study or refused to participate in it, the final sample was of a total of 165 participants (Figure 1).

Every single one of the TUs analyzed was established as an independent cluster, and it was assigned randomly to the CG (2 TUs), the EG1 (2 TUs), or the EG2 (2 TUs). Participants’ selection was performed in each TU and was also stratified by type of participant (66 mentors and 66 resident intern specialists).

### 2.5. Blinding Strategy

Due to the intervention’s nature, it was not possible for the participants to be blinded. However, to prevent possible cross-contamination between groups, different strategies were used to obtain the highest possible level of blindness. The person who performed the training sessions in the EGs was different from the investigator who conducted the evaluation visits. In addition, both the researcher who conducted the evaluation visits and the one who carried out the statistical data analysis were blinded to the group to which the participants belonged. Clear guidelines were administered to all participants in order not to reveal, along the assessment sessions, the group to which each TU had been randomly assigned.

### 2.6. Interventions

EG1 and EG2 training programs were based on the Jon Kabat-Zinn’s MBSR training program developed at the University of Massachusetts Medical Center [49,50]. Mindful self-compassion (MSC) program practices were also incorporated to complement the interventions [51,52,53]. The different sessions of each group were adapted to the characteristics of the health care professionals and differed in terms of the duration and time dedicated to the exercises [49,50]. EG1 participants belonged to an abbreviated training program, which consisted of 4 weekly sessions lasting 150 min and 15 min of daily practices at home. On the other hand, a standard MBSR program, consisting of 8 (one per week) sessions of 150 min with the daily practice for 30 min at home, was applied to the EG2 participants. All sessions were carried out in groups, looking for their practical application in the participants’ personal and/or professional field. To this purpose, moments of collective exploration were alternated with other silent moments as the best systems for dealing with difficult and complex situations. Power of emotions, perceived reality, knowledge of mindfulness, tolerance to emotional tension and stress, resilience, use of mindful communication, one-self-care, the management of time, and mindfulness integration into everyday life were some of the aspects that were promoted in the different sessions. The developed activities in each session were detailed in the study protocol previously [44]. Both training programs were incorporated in the TU and they were developed by the same accredited instructors. The therapist collected in a manual the standardized and uniform methodological criteria to follow, in order to make any variability associated with the instructors avoided. The participants in the CG received no intervention, but they attended to a 1 h information session, where the researchers explained their part in the research and they were invited to fulfill the assessment both in the pre- and post-test, coinciding with EG2. Participants agreed not to receive any intervention and not to participate in the activities of mindfulness training sessions or meditation techniques while the study was conducted. Once the field work was finished, they were offered the chance to benefit from a shortened training program.

### 2.7. Main Outcomes—Instruments

Participant’s anxiety and depression were the principal outcomes of the research, so, they were evaluated both in the pre-test, follow-up, and post-test evaluation sessions.

Psychological symptoms of depression and anxiety were measured with the GADS [54]. Two subscales make up this test: one for the detection of anxiety (Goldberg Anxiety Scale, GAS) and the other for the detection of depression (Goldberg Depression Scale, GDS). Each subscale contains nine dichotomous questions answered by yes/no; where the first four are mandatory, while the lasting five are only answered only in the case of any of the earlier ones are affirmative. The evaluator asks the participant for the different symptoms included in the GDAS, alluding to the prior 15 days. For each affirmative answer 1 point is added, not scoring in case of the negative one. The score of each subscale ranges from 0 to 9, while the GDAS from 0 to 18. When there is an affirmative answer in four or more GAS’ items, the person is considered to have anxiety; regarding depression, if the person gives two or more affirmative answers on the GDS it is considered that they have depression [54,55,56]. Montón Franco et al. validated GADS in Spanish population; it showed an adequate level on internal consistency with a Cronbach’s alpha of 0.81 for GADS, 0.74 for GAS, and 0.70 for GDS [57].

The participants’ attendance to person-to-person sessions was monitored so that the adherence to the training program could be measured. In addition, the participants had to write in a daily personal notebook or journal whether they did the exercises at home and show to the instructor in each session for supervision. All data of the participants that held an adequate adherence level were include in the statistical analysis. For this, it was considered that those participants had assisted to at least 3 of the 4 sessions in EG1 and 6 of the 8 sessions in EG2 had had an adequate adherence.

During the pre-test evaluation, information about the social and demographic variables, like age, sex, job category (nurse or physician), type of participant (mentor or internal resident specialist), workplace (clinic or hospital), time that they had been working in the SNHS, was also collected to evaluate potential predictor or confounding effects. A more detailed description of these variables can be found in the clinical trial protocol [44].

### 2.8. Procedure for Data Collection, Management and Monitoring

In the evaluation and the follow-up assessment visits, the measurement and data collection were conducted by same researcher, who had been specifically trained for these tasks previously. Another member of the research team carried out the randomization process, different from the one responsible for the later statistical data analysis. Each participant was assigned a unique alphanumeric code to make easier the identification of the data collected in the different evaluations within the study. This code consisted of 6 numbers, which corresponded to the participant’s date of birth (day/month/year in dd/mm/yy format), as well as the first two initial letters of their name and surname. A database was created, which only researchers from the investigation group involved in the statistical analysis had access. All the questionnaires used a double data entry procedure in order to keep the lowest error rate. The principal researcher was the responsible for adapting the procedures to the protocol and performing the weekly study monitoring and the database cleaning and debugging.

### 2.9. Ethical Considerations

This research’ protocol was accepted by the Clinical Research Ethics Committee of the Reina Sofía University Hospital in Córdoba (Spain) with reference 3845. Before entering the study, all participants knew the objective and its possible risks and benefits. The written and signed declaration of consent was granted by each person in accordance with the Declaration of Helsinki recommendations. The data collected were not used for purposes different than those exposed in the written declaration of consent or was passed on to others outside the investigation. The participants’ data confidentiality was guaranteed in accordance with the Organic Law 3/2018 of 5 December, on the Protection of Personal Data and Guarantee of Digital Rights (Law 14/2007 of 3 July), on Biomedical Research, and Regulation 2016/679 of the European Parliament and of the Council of 27 April 2016, on the General Data Protection of Natural Persons in the Processing of Personal and Free Circulation of these data.

### 2.10. Statistical Analysis

The results were analyzed on an intent-to-treat basis, in order to copy with withdrawals, losses, deviation from the protocol, or anything that happened after randomization. Continuous variables were expressed in mean and SD, while categorical variables were summarized as frequencies distribution and percentages. In continuous variables, the Kolmogorov–Smirnov test was used in order to verify the normality criteria; the Runs test was used to contrast the assumption of randomization; and the Levene test was used to evaluate the homoscedasticity’s equality. In all cases, *p* > 0.05 was observed, being justified the use of parametric tests. The comparability between the three groups in the pre-test evaluation, in terms of sociodemographic variables, was assessed with chi-squared test or Student’s *t* test for independent samples. The one-way variance analysis (ANOVA) test was used to analyze the MBRS training program effect on the different study groups participants’ anxiety and depression. To compare the mean anxiety and depression scores in each group over time, a repeated measures ANOVA test was performed. The absence or presence of sphericity was calculated using the Mauchly’s W test, performing the Greenhouse–Geisser correction when necessary. Multiple comparisons were performed with the Bonferroni correction. The squared eta coefficient (η^2^) was calculated for the estimation of interventions’ effect side on the anxiety and depression levels; the results were interpreted in consonance with these criteria: if 0 ≤ η^2^ < 0.05, no effect; if 0.05 ≤ η^2^ < 0.26, minimal effect; if 0.26 ≤ η^2^ < 0.64, moderate effect; and if η^2^ ≥ 0.64, strong effect [58]. Finally, in order to eliminate the effect that could be attributable to variables that were not included in this research’ design and to analyze the effects of the intervention, the changes between the CG and EG in post-test and follow-up punctuations were compared by a covariance analysis test (ANCOVA), with the pre-test scores of the dependent variables as covariate and the intervention groups as a fixed factor. The contrasting hypothesis established as the limit of statistical significance an alpha risk of 0.05. The data were analyzed using the SPSS Statistics software for Windows, version 25.0 (IBM SPSS Inc., Chicago, IL, USA) and MLwiN software, version 3.0 (Center for Multilevel Modelling, University of Bristol, Bristol, UK, 2019).

## 3. Results

### 3.1. Baseline Aspects of the Participants in this Research

Out of the 165 participants that were included in the study (63 in CG, 39 in EG1, and 63 in EG2) there were 38 losses due to the refusal to a continuous participation in the research and 15 because due to an inappropriate program adherence. This dropout rate was higher in EG2 (*n* = 26) than in EG1 (*n* = 15), being the main reason a refusal to continue to participate in the program. As a result, 112 participants completely finished the research and were included for the analysis of the data, 51 belonged to the CG, 24 to the EG1, and 37 to the EG2. (Figure 1).

Table 1 shows the participants’ baseline social and demographic characteristics related to the study group. Most of the participants were women (*n* = 86, 76.79%), and the mean age of the sample was 40.61 years (SD ± 12.61); 84.82% of participants (*n* = 95) were Primary Care workers, being the physician the most represented category of professionals (*n* = 95; 84.82%). The mean time that they had been working was 12.88 (SD ± 13.15) years. The sample showed a uniform distribution uniformly to mentors and resident intern specialist (62 versus 50). In the initial evaluation, statistically significant differences between the three groups were found regarding work experience, job category and age.

### 3.2. Anxiety and Depression

In baseline evaluations, it was found no statistically significant differences between the CG, EG1, and EG2 in the GADS (*p* = 0.500), GAS (*p* = 0.495), and GDS (*p* = 0.615), which indicated the equivalence between the groups before starting the MBRS training programs. However, when comparing the scores of the post-test evaluation, statistically significant differences were observed in the GADS and GDS, with a weak effect size (η^2^ = 0.079 and η^2^ = 0.114, respectively). In both cases, these differences were observed between CG and EG2, with higher mean scores in the first of them, according to the results of the pairwise comparisons by Bonferroni test. Likewise, in the follow-up evaluations, the differences in the mean scores of the GADS, GAS, and GDS were also statistically significant, with a weak effect size (η^2^ < 0.26). The CG participants showed higher mean scores in these three variables with respect to those of EG2 (Table 2).

In the intragroup comparisons, a significant reduction in the mean scores of the post-test evaluation in relation to those of the pre-test in the GADS and GDS was observed in the subjects that attended to the standard program, with significant and minimal effect sizes (η^2^ < 0.26). However, no statistically significant differences were obtained in the mean anxiety and depression scores at the follow-up evaluation in relation to pre or post-test one (Table 3).

Table 4 summarizes the comparison between CG, EG1, and EG2 in the post-test and follow-up punctuations, with pre-test scores control, using ANCOVA. This analysis showed statistically significant differences in the GADS and GDS variables, between different groups, results that confirm the previous intergroup comparisons. Thus, differences, primarily in EG2, may be ascribed to the MBRS training program (Table 4).

## 4. Discussion

In this research, a standard MBRS program effects among mentors and resident intern specialists of FCMN were examined; these effects were also compared to those of another abbreviated one. The potential benefits of both training programs on anxiety and depression were been studied. The main results showed an improvement in the GADS and GDS scores in the participants who attended to the standard program, with the effects’ maintenance over time. However, the abbreviated training program on anxiety and depression levels showed no significant impact.

MBRS training programs have proven to be very useful in emotional regulation improvement; and to the depletion of anxiety and depression levels, and post-traumatic stress disorders [59,60,61], characteristic symptoms during the COVID-19 pandemic, especially among health care workers [33]. Several studies have also shown an increase in the consciousness level, an improvement in coping strategies in times of stress, a greater emotion control, and a significant anxiety and depression levels reduction when these interventions were performed [62,63,64]. Goyal et al. completed a systematic review of 47 clinical trials, where the aim was to analyze mindfulness and meditation training programs efficacy on stress in the general population, and concluded that these techniques gave moderate evidence of improved anxiety at 8 weeks (η^2^ = 0.38; 95% confidence interval (CI) 0.12–0.64) and at 3–6 months (η^2^ = 0.22; 95% CI 0.02–0.43), as well as depression at 8 weeks (η^2^ = 0.30; 95% CI 0.00–0.59) and at 3–6 months (η^2^ = 0.23; 95% CI 0.05–0.42) [27]. On the other hand, a 38 randomized clinical trial meta-analysis performed by Spinelli et al. focused on the effect of mindfulness activities on qualified and trained health care professionals [65]. In this review it was highlighted that mindfulness training program helped significantly to reduce anxiety (Hedge’s g = 0.47; 95% CI 0.27–0.67) and depression (Hedge’s g = 0.41; 95% CI 0.26–0.57) at post-intervention.

Most of the research to date has demonstrated the multiple benefits of standard 8-week MBRS programs on healthcare professionals [23,65]. Some authors have analyzed these training programs’ effectiveness when implemented in a shorter period of time, such as 4 or 3 weeks [50,66,67,68,69]. In all cases, a significant reduction in anxiety and depression levels was observed, maintaining their effects at 9 months follow-up [50]. However, the number of studies comparing the effectiveness of abbreviated and standard interventions in health professional is very limited. De Marzo et al. compared the effectiveness of an 8-week MBRS training program and a 4-week-abbreviated version for well-being improvement in a sample of Health Sciences undergraduate students, and concluded that both types of programs worked in the same way [70]. Kriakus et al. performed a meta-analysis with the aim to update the latest data related to the efficacy of mindfulness practice among health care professionals, additional evidence was provided that these programs were effective in improving psychological aspects, anxiety, and depression levels [23]. Furthermore, a standard 8-week training program showed the same effectiveness as an abbreviated 4-week one when decreasing these symptoms. The findings of this study revealed that only the standard training program improves the depression levels, with the maintenance of its effects at the 3-month follow-up.

In this research, no improvements in anxiety levels were observed in either of the two EGs. Consistent with these findings, two clinical controlled trials reported non-significant effects in anxiety [71,72]. These results do not coincide with those obtained by other authors, who have demonstrated the positive effects of an intervention based on mindfulness in minimizing anxiety symptoms. Irving et al., completed a systematic re-view in which the important benefits of MBSR training program on the improvement of well-being and stress resistance were examined amongst healthcare professionals and concluded that these types of interventions reduced anxiety [73]. In a quasi-experimental trial, Barbosa et al. analyzed the impact of the 8-week MBSR training program on students from five healthcare degrees and observed a significant decline in anxiety levels at weeks 8 and 11 compared with initial evaluation [74]. In another study, a sample of primary care physicians participated in an abbreviated MBRS training program, improving their indicators of anxiety [50].

In the same line with the results of the present research, different researchers have concluded that the standard MBRS training program can reduce the depressive symptoms of the participants. A randomized controlled trial by Song et al., examined the effects of an 8-week MBRS program on depression levels in a sample of 50 Korean nursing students, and showed a significantly greater decreases in depression measures [75]. In the same way, Pizutti et al. evaluated the Breathworks’ Mindfulness for Stress 8-week training program effects on psychiatric and depressive symptoms and negative effects in 84 primary healthcare professionals, and observed a statistically significant depletion in all of the variables analyzed after the intervention [76].

In this kind of training program, the effects that are achieved in the short term must be considered with the same importance as their maintenance along time. Fortney et al., observed the continuity of significant reductions in depression levels after a 9-month follow-up [50]. In the study by Lane et al., the mindfulness training program not only reduced negative symptoms such as depression in a sample of 200 subjects with different professions, but its benefits were maintained over time 3 months after the intervention had already ended [40]. These results are in line with those obtained in this study, since the decrease in depression levels obtained in EG2 in the post-test were maintained during the three subsequent months.

In order to objectively analyze the psycho-physiological effects of MBSR and assess the changes pre- and post-intervention, different studies have suggested the use of the event-related brain potentials or EEG [33,77].

This sub-analysis is part of a global project in which variables such as levels of mindfulness, self-compassion, and self-perceived empathy in these professionals were also analyzed. However, unlike the results obtained in the levels of anxiety and depression, it was observed that the abbreviated program of 4 weeks did not produce improvements on the mentioned aspects; although, the standard program of 8 weeks did allow improvements in mindfulness and self-pity to be obtained [78].

The present study is innovator in the comparison of the effects of an abbreviated MBSR and MSC training program with a standard one on anxiety and depression levels in mentors and resident intern specialists in Spain. However, these findings must be taken into account among the context of their strengths and limitations. Among the principal strengths, there were: the possibility of determining the causal relationship between variables through the longitudinal methodology; the guarantee of obtaining valid information and reducing the probability of information biases thanks to the use of instruments that were validated for the Spanish population; the baseline anxiety and depression levels were equivalent so that all participants were considered to start from a similar situation, as well as the evaluation of the continuity of the effect over time. Furthermore, this research also has certain limitations that may have influenced the obtained results, and decreased its representativeness. Despite the random assignment of the TUs to the different groups so that the contamination risk could be minimized, statistically significant differences were found between the three groups related to age, job category, and time that they had been working in the SNHS. In addition, the final sample size was inferior to that previously calculated at the beginning due to the COVID-19 pandemic epidemiological situation, which may have affected to the results obtained. This aspect must be into account in future research. On the other hand, the dropout rate due to refusal to continue participating in the program was high, being more pronounced in GE2 than in GE1. This could be explained by the differences in the sociodemographic characteristics of the participants between GE1 and GE2 in relation to the years of experience working and in the professional category. Because the EG participants had fewer years of experience, prevailing residents over tutors, the opposite occurring in the EG1. These characteristics could have influenced the motivation of the participants, which will be taken into account for future research. An analysis based on an intention-to-treat was carried out as the characteristics of the people that did not respond differ from those of the respondents, and therefore can control for this selection bias. Although a representative sample of Spanish mentors and general practitioners was available in FCMN, the overrepresentation of primary care workers, women, and physicians reduced the study results generalizability. Although the CG participants were not offered a theoretical practical session of either mindfulness or meditation, it could not be guaranteed that they would remain inactive during the period in which the study was carried out to evaluate the expected results’ differences when comparing this group with the EG.

## 5. Conclusions

An 8-week MBSR training program, aimed at tutors and resident specialists in FCMN, produced a significant improvement in levels of anxiety and depression in general, and depression in particular, maintaining these effects for three months. However, a 4-week version was not associated with significant changes in anxiety or depression levels. More representative and larger investigations must be conducted to support the effectiveness of abbreviated MBSR and MSC programs for primary care professionals, which could also increase adherence and accessibility to such training.

## Figures and Tables

**Table 1:** Baseline participants’ aspects.

Variable	Total *n* = 112	CG *n* = 51	EG1 *n* = 24	EG2 *n* = 37	*p*-Value
Age (in years)	41.61 ± 12.61	40.34 ± 13.22	47.66 ± 13.67	35.73 ± 12.04	<0.001
Sex					
Male	26 (23.21)	11 (21.57)	6 (25.00)	9 (24.32)	0.978
Female	86 (76.79)	40 (78.43)	18 (75.00)	28 (75.68)	
Occupation					
Physician/medicine	95 (84.82)	41 (80.39)	20 (83.33)	34 (91.89)	0.165
Nurse	17 (15.18)	10 (19.61)	4 (16.67)	3 (8.11)	
Professional type					
Tutor	50 (44.64)	24 (47.06)	15 (62.50)	11 (29.73)	<0.001
Resident	62 (55.36)	27 (52.94)	9 (37.50)	26 (70.27)	
Workplace					
Health Center	95 (84.82)	40 (78.43)	22 (91.67)	33 (89.19)	0.217
Hospital	17 (15.18)	11 (21.57)	2 (8.33)	4 (10.81)	
Work experience (years)	12.88 ± 13.15	13.13 ± 12.95	19.49 ± 13.91	8.91 ± 11.06	<0.001

Values expressed in mean ± standard deviation or frequencies (percentages). Abbreviations: CG: Control Group; EG1: Experimental Group, 4 weeks; EG2; Experimental Group, 8 weeks.

**Table 2:** Inter-group comparison of GADS, GAS, and GDS at different evaluation moments. One-way ANOVA.

Group	Assessment	CG		EG1		EG2		F	*p*-Value	η^2^
		Mean	SD	Mean	SD	Mean	SD			
GADS	Pre-test	8.20	4.28	7.10	5.20	7.68	4.55	0.697	0.500	0.009
	Post-test	7.82 *	4.64	5.82	5.51	4.82 *	3.88	5.227	0.007	0.079
	Follow-up	8.35 *	4.19	6.41	5.63	5.18 *	3.72	5.725	0.004	0.095
GAS	Pre-test	5.20	2.59	4.53	2.99	4.96	2.77	0.705	0.496	0.009
	Post-test	4.64	2.68	3.57	2.94	3.46	2.70	2.645	0.075	0.042
	Follow-up	5.15 *	2.41	4.12	3.08	3.54 *	2.57	4.238	0.017	0.072
GDS	Pre-test	3.00	2.27	2.56	2.56	2.71	2.13	0.487	0.615	0.006
	Post-test	3.17 *	2.35	2.25	2.81	1.36 *	1.51	7.823	0.001	0.114
	Follow-up	3.19 *	2.28	2.29	2.88	1.64 *	1.60	5.252	0.007	0.088

* *p*-value < 0.05 in post-hoc Analysis (Bonferroni test) between CG and EG2. Abbreviations. CG: Control Group; EG1: Experimental Group, 4 weeks; EG2: Experimental Group, 8 weeks; SD: Standard deviation; GADS: Goldberg Anxiety and Depression Scale; GAS: Goldberg Anxiety Scale; GDS: Goldberg Depression Scale.

**Table 3:** Intra-group comparison of GADS, GAS, and GDS at the same evaluation moment. ANOVA for repeated measures.

Variable	Group	Pre-Test		Post-Test		Follow-Up		MS	F	*p*-Value	η^2^
		Mean	SD	Mean	SD	Mean	SD				
GADS	CG	8.20	4.28	7.82	4.64	8.35	4.19	6.131	0.806	0.451	0.025
	EG1	7.10	5.20	5.82	5.51	6.41	5.63	7.314	0.850	0.437	0.050
	EG2	7.68 ^$^	4.55	4.82 ^$^	3.88	5.18	3.72	33.722	3.224	0.040	0.123
GAS	CG	5.20	2.59	4.64	2.68	5.15	2.41	1.939	0.750	0.476	0.023
	EG1	4.53	2.99	3.57	2.94	4.12	3.08	4.843	1.120	0.339	0.065
	EG2	4.96	2.77	3.46	2.70	3.54	2.57	10.597	2.194	0.123	0.087
GDS	CG	3.00	2.27	3.17	2.35	3.19	2.28	1.768	0.752	0.476	0.023
	EG1	2.56	2.56	2.25	2.81	2.29	2.88	0.961	0.622	0.543	0.037
	EG2	2.71 ^$^	2.13	1.36 ^$^	1.51	1.64	1.60	6.514	3.583	0.036	0.135

^$^*p*-value < 0.05 in post-hoc Analysis (Bonferroni test) between pre-test and post-test. Abbreviations. SD: Standard deviation; MS: Mean Square; CG: Control Group; EG1: Experimental Group, 4 weeks; EG2: Experimental Group, 8 weeks; GADS: Goldberg Anxiety and Depression Scale; GAS: Goldberg Anxiety Scale; GDS: Goldberg Depression Scale.

**Table 4:** Comparison between groups: post-test–follow-up punctuations with pre-test scores control. ANCOVA.

Evaluation	Variable	Source	Type III Sum of Square	df	MS	F	*p*-Value	η^2^
Post-test	GADS	Pre-test GADS	656.61	1	656.61	49.880	<0.001	0.354
		CG/EG1/EG2	118.15	2	59.08	4.488	0.014	0.090
		Error	1197.91	91	13.16			
	GAS	Pre-test GAS	195.23	1	195.23	37.249	<0.001	0.388
		CG/EG1/EG2	24.53	2	12.26	2.340	0.102	0.128
		Error	476.95	91	5.24			
	GDS	Pre-test GDS	166.40	1	166.40	57.660	<0.001	0.388
		CG/EG1/EG2	38.40	2	1.92	6.653	0.002	0.128
		Error	262.62	91	2.87			
Follow-up	GADS	Pre-test GADS	413.79	1	413.80	29.497	<0.001	0.272
		CG/EG1/EG2	85.04	2	42.52	3.031	0.050	0.071
		Error	1108.26	79	14.03			
	GAS	Pre-test GAS	107.98	1	107.98	18.644	< 0.001	0.191
		CG/EG1/EG2	25.78	2	12.89	2.226	0.115	0.053
		Error	457.52	79	5.79			
	GDS	Pre-test GDS	108.94	1	108.94	32.063	<0.001	0.289
		CG/EG1/EG2	19.53	2	9.77	2.874	0.049	0.068
		Error	268.42	79	3.40			

Abbreviations. df: Degrees of Freedom; SD: Standard deviation; MS: Mean Square; CG: Control Group; EG1: Experimental Group, 4 weeks; EG2: Experimental Group, 8 weeks; GADS: Goldberg Anxiety and Depression Scale; GAS: Goldberg Anxiety Scale; GDS: Goldberg Depression Scale.
